# A Modified Transgluteal Approach Sparing Abductor Function in Total Hip Arthroplasty Results in a Low Postoperative Dislocation Rate: A Retrospective Study of Short- and Long-Term Outcomes

**DOI:** 10.7759/cureus.73804

**Published:** 2024-11-16

**Authors:** Michael H Ward, Akshay Date, Tien Yeoh, Patrick Li

**Affiliations:** 1 Orthopaedics and Trauma, King's College London, London, GBR; 2 Orthopaedics, King's College Hospital, London, GBR; 3 Orthopaedics and Trauma, King's College Hospital, London, GBR

**Keywords:** adult, arthroplasty, hip/adverse effects, postoperative complications, replacement, treatment outcomes

## Abstract

Background

A modified transgluteal approach in total hip arthroplasty (THA) can be utilized to preserve abductor muscle function and reduce dislocation rate. We present a study evaluating outcomes for a modified transgluteal approach using a validated patient-reported outcome measure (PROM) tool, the Oxford Hip Score (OHS).

Methods

This was a retrospective single-centre study over a four-year period. Short-term data was collected including intraoperative and postoperative complications, length of stay (LOS) in the hospital, and time from operation to mobilising independently. One year of data was collected, including plain radiograph findings and the incidence of Trendelenburg gait. Patients were contacted at a mean time of 2.7 years post-operatively so that OHS could be conducted.

Results

A total of 100 patients were identified within the inclusion criteria. The mean LOS for all patients was 2.8 days. The mean time from operation to mobilising independently without walking aids was 4.9 weeks. At the one-year follow-up, there was satisfactory radiographic assessment in 100% of patients. The mean OHS was 45.5 at 2.7 years, indicating satisfactory joint function in all patients.

Conclusion

This study supports the use of the modified transgluteal approach in THA, showing favourable outcomes in the time from operation to cessation in the use of walking aids, LOS and OHS. We report zero cases of Trendelenburg gait at the one-year follow-up and no dislocations at the three-year follow-up. Further studies are required to compare the outcomes of TGA to other approaches in THA.

## Introduction

Total hip arthroplasty (THA) is a procedure which has reliable and successful outcomes, with demand predicted to increase by 40% over the next 40 years [1]. Still, there remains no consensus on the impact of the surgical approach used in THA on patient outcomes. Commonly performed surgical approaches to the hip include the posterior, direct lateral, anterolateral and direct anterior approach, each with its own advantages and disadvantages [2,3]. Although there are numerous level-one studies in the literature comparing THA direct anterior versus posterior approach, there remains a paucity of evidence looking at outcomes from the anterolateral approach (ALA) in THA [4,5].

Herein, we report the results of a modified transgluteal approach to the hip which retains the advantage of a low dislocation rate traditionally ascribed to anterolateral approaches yet preserves abductor muscle function.

The ALA approach to the hip, originally described by Watson-Jones in 1936, utilises the interval plane between tensor fascia lata and gluteus medius [6]. Although originally described as muscle-sparing, multiple variations of this approach have been described, most involving detachment of the abductors to some degree. The modified ALA allows adequate visualisation of the acetabulum and proximal femur, which is useful in both primary and revision THA. There is some evidence to suggest that detachment and repair of the abductor muscles may lead to poorer functional outcomes, with some studies showing abductor-sparing approaches to have a lower incidence of post-operative Trendelenburg gait [7,8]. However, the literature also suggests the modified ALA may have lower dislocation rates compared to the posterior approach in THA [9].

We present a retrospective study looking at outcomes, in particular the incidence of Trendelenburg gait and dislocation, from a cohort of patients who underwent THA surgery using a modified transgluteal approach. We describe the approach and evaluate outcomes using a validated [10] patient-reported outcome measure (PROM) tool, the Oxford Hip Score (OHS).

## Materials and methods

This study was carried out at a single institution (King's College Hospital, London, United Kingdom). Patients who underwent elective primary THA surgery under the care of a single surgeon using the modified transgluteal approach between October 2019 and September 2023 were identified retrospectively. Exclusion criteria were revision THA and THA performed for trauma. Data was collected on patient age, gender, right or left side surgery and type of surgery performed (cemented, uncemented or hybrid). Short-term data was collected including intraoperative complications, length of stay (LOS) in hospital and post-operative complications. One year of data was collected; longer-term complications, plain radiograph findings and clinic letters were analysed for the presence or absence of Trendelenburg gait. The data was analysed to calculate the incidence of short-term and medium-term complications subdivision of complications by type. Patients were contacted via telephone after a mean time of approximately three years post-operatively to conduct an OHS questionnaire. The OHS was performed by a single author using a scripted online auto-calculator tool. At the end of the questionnaire, patients were asked if they had any post-operative dislocations since the documented one-year clinic follow-up. As this was a retrospective observational study, we did not seek ethical approval. All patients involved in this study consented to the collection, presentation and publication of their data. All intra-operative photographs taken to demonstrate the approach are reproduced with the patients' written consent for presentation and publication. 

Surgical approach

The patient wass placed into a lateral decubitus position with the ipsilateral hip facing up and hip stabilisers installed on the operating table. A sturdy position was maintained so that the pelvis did not move intra-operatively. The ipsilateral limb was then prepared and draped.

**Figure 1:**
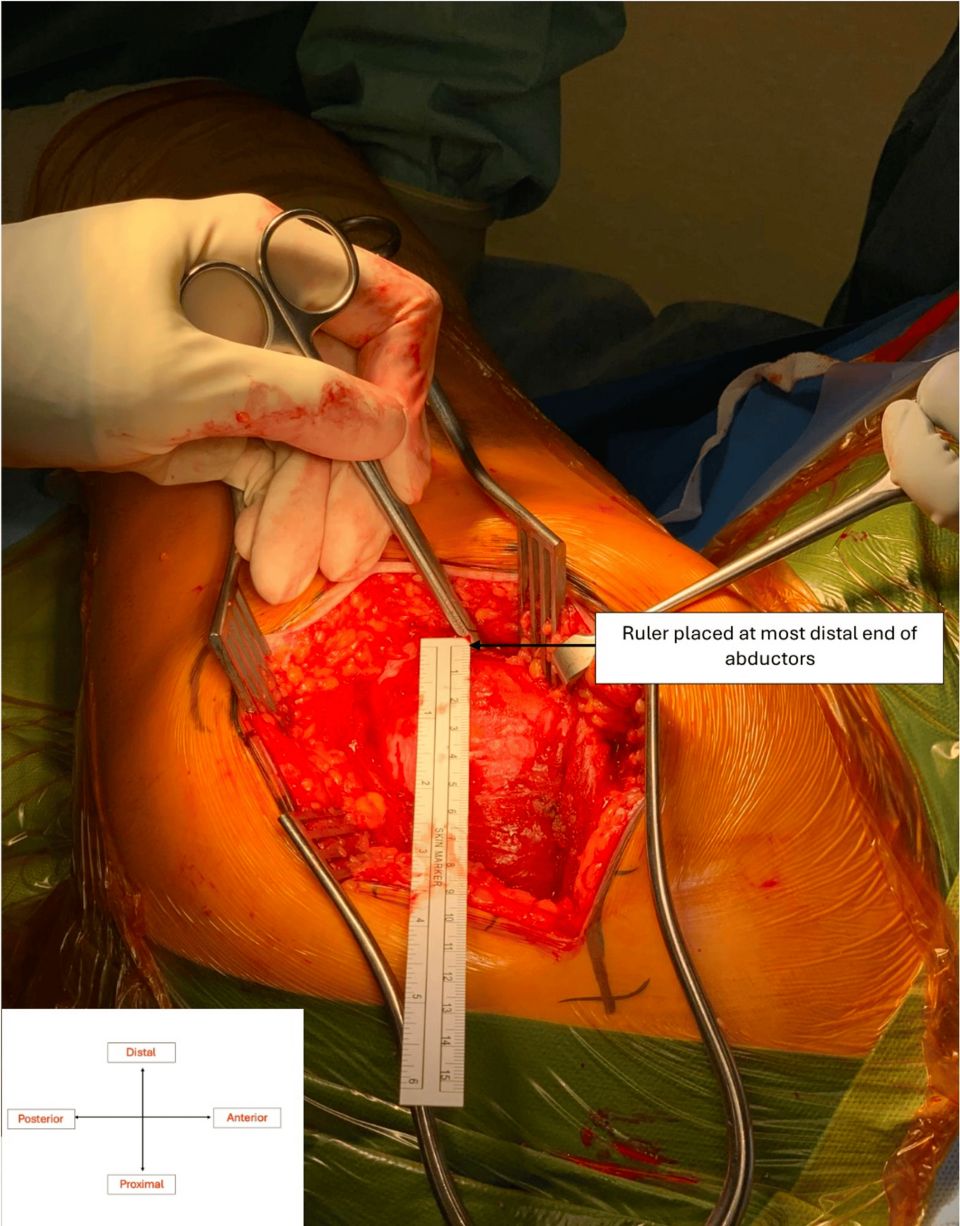
Distal end of abductors marked with McIndoe scissors and ruler used to outline 1cm from the distal end of abductors Original intra-operative imaging taken with the consent of the patient.

**Figure 2:**
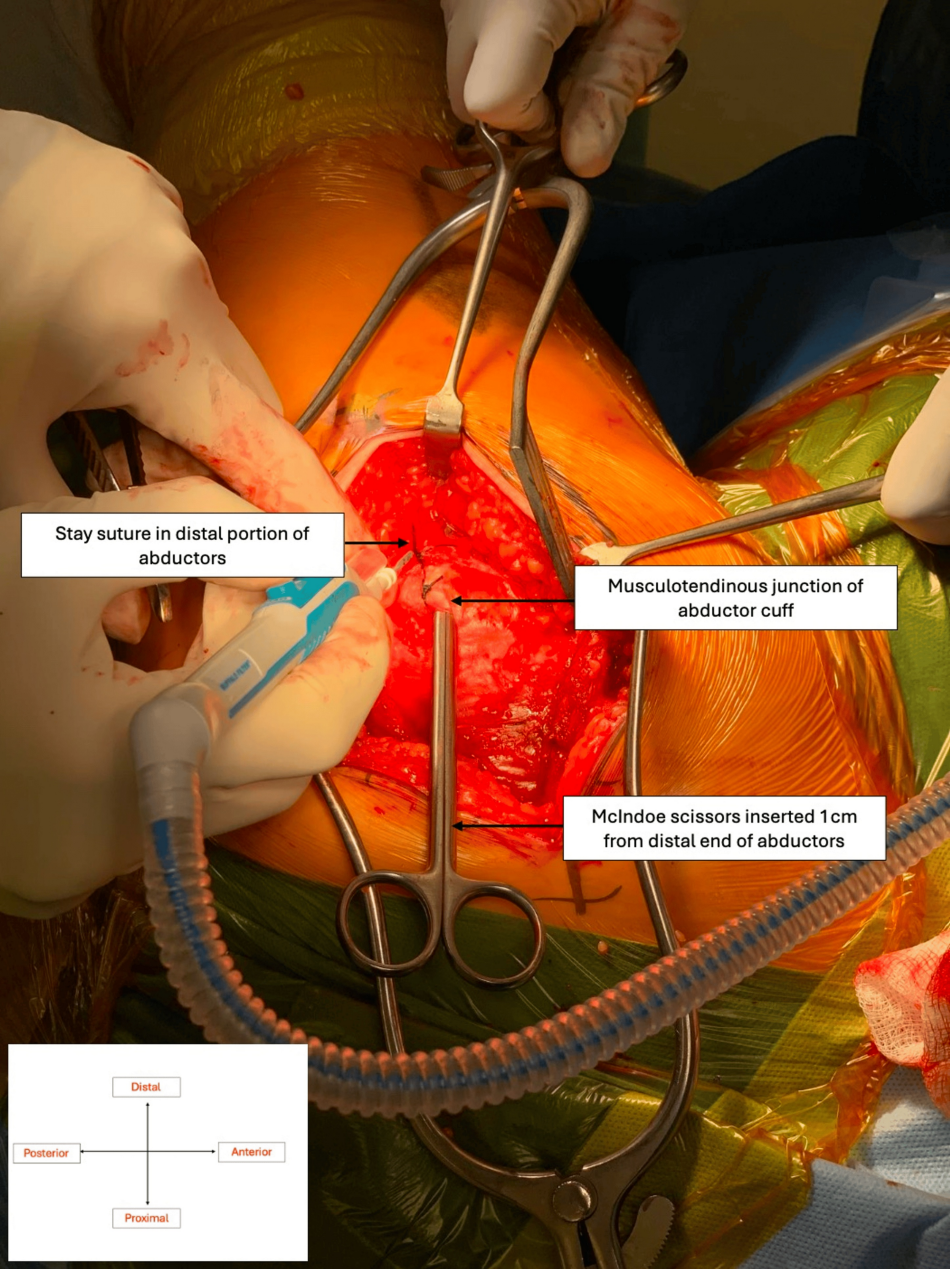
McIndoe scissors marking 1 cm proximal from abductor edge with stay sutures (purple) sited in abductor cuff at its musculo-tendinous junction Original intra-operative imaging taken with the consent of the patient.

**Figure 3:**
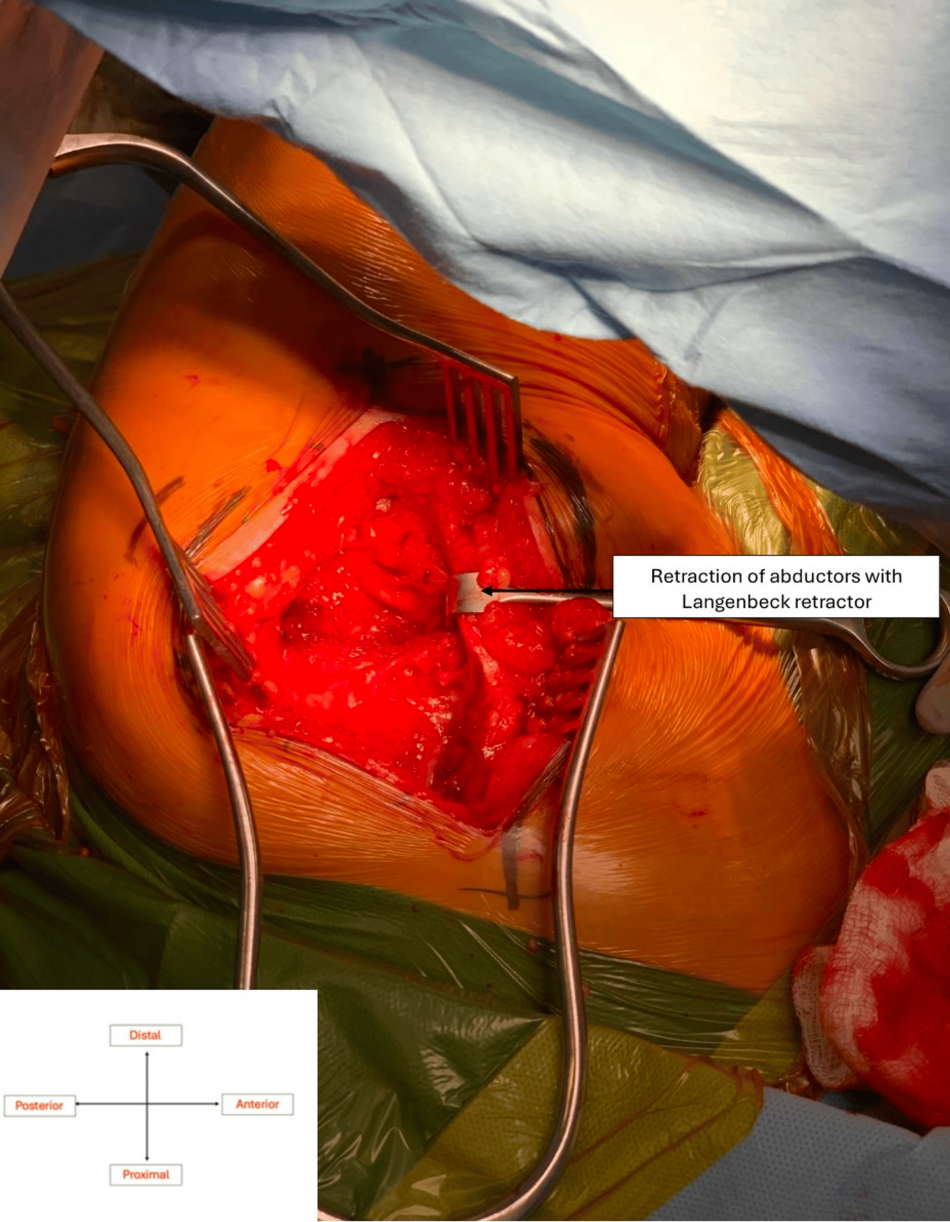
Retraction of abductors to allow capsulotomy Original intra-operative imaging taken with the consent of the patient.

**Figure 4:**
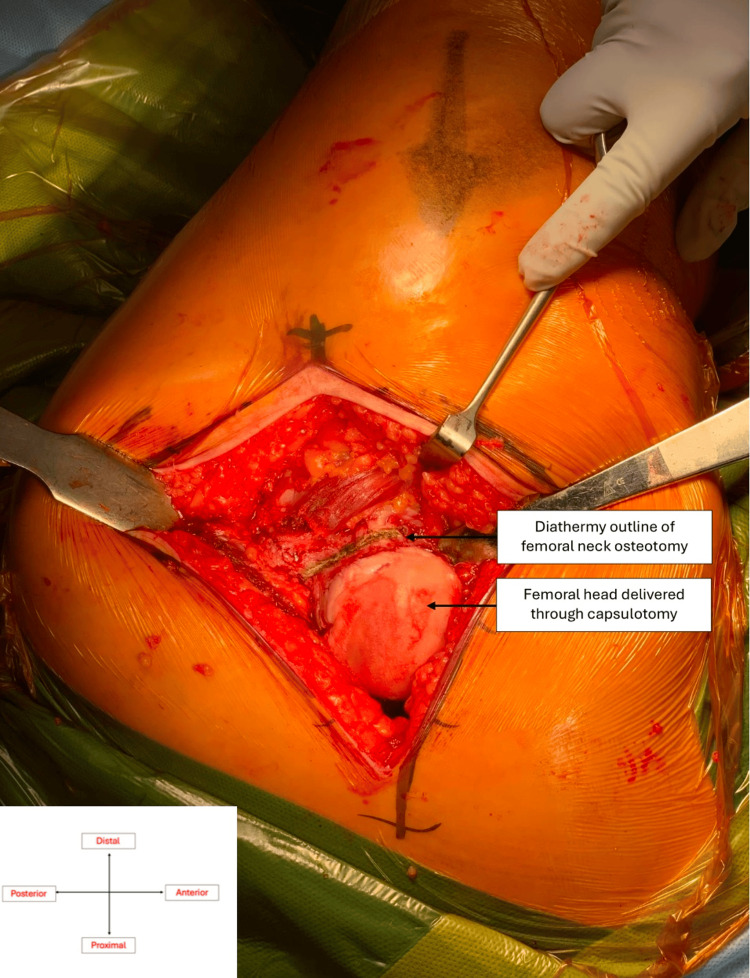
Delivery of femoral head with diathermy mark for femoral neck osteotomy Original intra-operative imaging taken with the consent of the patient.

**Figure 5:**
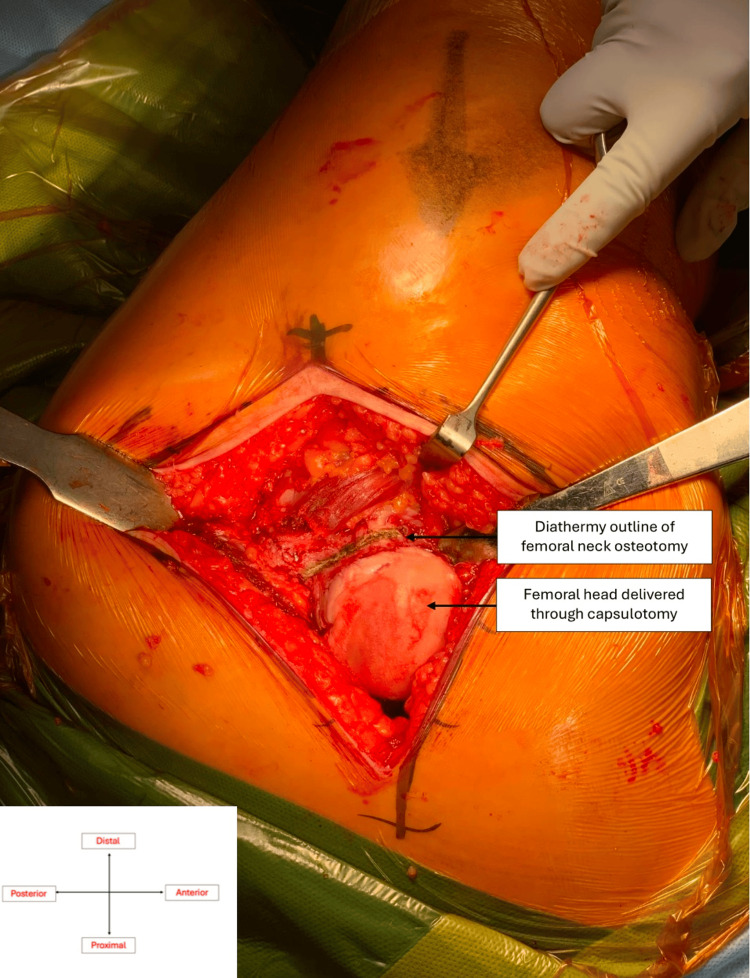
View of left acetabulum following osteotomy of femoral neck Original intra-operative imaging taken with the consent of the patient.

The incision was centred on the greater trochanter in a straight line, angled slightly posteriorly superior to the greater trochanter and slightly anteriorly inferior to it. The dissection proceeded through the fat layer and then the gluteal fascia and fascia lata. The trochanteric bursa was incised to expose the gluteus medius muscle and tendon. The lowermost 1-2 cm of the abductor cuff, comprised solely of horizontal fibres, was separated from the broad bulk of the abductor complex using McIndoe dissecting scissors. None of the oblique fibres with a vertical component were taken. A 1-Vicryl stay stitch was placed at the musculotendinous junction of the distal 1-2 cm of the cuff and this is incised (Figures 1-2), leaving a healthy cuff distally for subsequent repair. The inferior cuff of the combined gluteus medius and minimus muscle was then elevated as one structure from the underlying hip capsule. The superior gluteal nerve was not at risk because of the small size of the flap (1-2 cm). Having exposed the antero-inferior capsule of the hip, the antero-superior and superior capsule were freed from the bulk of the abductors by submuscular dissection, taking care not to cut any abductor muscle. This can be done easily because there is a clear plane between the muscle and the capsule. Once the capsule was exposed, the abductors were retracted to allow for capsulotomy (Figure 3) in line with the neck of the femur and then transversely, just medial to the intertrochanteric ridge, superiorly and inferiorly. The hip was dislocated by external rotation and the neck cut (Figure 4). Once the neck was cut, the acetabulum was exposed with the use of a Norfolk and Norwich retractor and an inferior retractor (Figure 5). Acetabular preparation is then completed followed by implantation. Finally, the leg was brought into flexion, external rotation, and adduction, allowing femoral preparation and implantation. Closure was straightforward with continuous absorbable sutures in layers. The stay stitch within the abductor cuff allowed solid and accurate repair of the cuff at the end of the procedure.

## Results

A total of 100 patients (male n=40, female n=60; mean age 67.5 years, range 47-86 years) were identified (Table 1). All patients underwent primary THA (right hip n=47, left hip n=53; hybrid n=73, uncemented n=19, cemented n=8) in a single institution between October 2019-September 2023. All patients were operated on by the same consultant surgeon with the modified transgluteal approach.

**Table 1:** Patient characteristics

	Male	Female
Patients	40	60

No complications were reported in 94% of patients (n=94). Of the 6% of patients (n=6) that had complications, two had superficial wound infections managed with antibiotics alone, one had a transient sciatic nerve neuropraxia with complete motor and near complete sensory resolution, one patient had a minor intraoperative calcar crack for which a prophylactic cable was positioned as a precaution and one patient had a DVT. One patient required revision surgery having sustained a fall whilst running two years post-operatively.

The mean inpatient LOS for all patients was 2.8 days (range 1-9 days). The mean time from the operation date to mobilising independently without walking aids was 4.9 weeks (range 1-28 weeks).

At one year follow-up there was satisfactory radiographic assessment with no wear, loosening, fracture, or prosthetic migration in 100% of patients. OHS phone questionnaires were performed at an average time of 971.9 days postoperatively (range 196-1645 days) with a mean score of 45.5 for all patients. Pre-operative OHS data, although recorded, was lost since the introduction of electronic patient records. There were no dislocations (0%) reported at the one- or three- year follow-ups and no Trendelenburg gait (0%) was observed at the mid-term follow-up by a dedicated musculoskeletal specialist physiotherapist.

## Discussion

There is a lack of consensus in the literature on the optimum surgical approach for THA, with various advantages and disadvantages of each approach documented. Our study highlights the merits of a transgluteal in THA that preserves abductor function and retains the advantage of a low dislocation rate.

OHS

Assessment of outcomes from orthopaedic interventions has become more patient-focused in recent years [11]. The OHS is a short, reproducible and validated PROM tool for evaluation of outcomes after THA [11,12]. The questionnaire consists of 12 questions, with scores ranging from 0 (most severe symptoms) to 48 (least severe). A score from 40-48 is considered to indicate satisfactory joint function [13]. We report a mean OHS of 45.46 at a mean of 2.7 years post-operatively, indicating high patient satisfaction at long-term follow-up. A multicentre prospective study analysed 1,035 patients undergoing THR through either an anterolateral or posterior approach, with postoperative OHS conducted at one, three and five years used as a primary outcome [14]. At five years, mean scores were 40.1 and 39.8 (adjusted for the new score out of 48 instead of 60) for the anterolateral and posterior approaches respectively, with no significant difference at three (p=0.256) or five years (p=0.329) between groups. Our study reported better OHS outcomes with the transgluteal approach compared to the OHS outcomes from that study. A second study randomised 75 patients to anterior and posterior approaches; they reported a mean OHS of 46 and 47 respectively at five years (p=0.93), both comparable to the results in our study [15]. Harris Hip Score (HHS) is an alternative PROM tool that is utilised in the literature. One prospective randomised control study of 123 patients evaluated HHS in patients undergoing THA via an anterior versus a transgluteal approach; there was no statistical difference (p=0.477) in scores at the one-year mark in either approach [16]. Findings from the literature suggest the approach used in THA has no statistical significance in the long-term outcomes of OHS or HHS [17].

LOS

Post-operative LOS has implications on patient psychological well-being, hospital-related complications (hospital-acquired infections) and an economic impact on resources. Intraoperative (blood loss) and postoperative (infection) factors have been shown to have a greater risk of prolonging LOS compared to preoperative variables such as patient comorbidities [18]. A retrospective study of 5,341 patients showed a mean LOS of 1.8 days for the direct anterior approach and 2.3 days for the posterior approach (p<0.001) [19]. A systematic review of 2010 patients also concluded that the direct anterior approach had a significantly shorter LOS compared to the posterior approach with a mean difference of 0.33 days (p=0.003) [20]. This is further supported by another systematic review and meta-analysis of 774 patients comparing THA performed by the anterior versus posterior approach analysing LOS as an outcome (p=0.002) [5]; the authors found eight studies (n= 314) looking at the posterior approach reporting a mean LOS of 2.82 days and another eight studies (n=319) with the anterior approach reporting a mean LOS of 2.57 days. The LOS figures from our study are comparable to the literature, with our patients having a mean LOS of 2.76 days. The literature strongly suggests direct anterior approach has a significantly shorter LOS than posterior-based approaches. Further work is required to compare the TGA to posterior and direct anterior approaches while looking at LOS.

Complications

The surgical approach in THA may have a crucial role in the development of certain complications [21]. A large retrospective study of 3574 patients compared complication rates for five surgical approaches with a mean follow-up time of 3.72 years; they reported the anterior approach had the highest complication rate (8.5%) and the posterior approach had the lowest complication rate (5.85%, p=0.006)[22]. Most complications were deep infection and periprosthetic fractures; the dislocation rate was 0.84% and 1.28% for the posterior and anterior approaches, respectively. Results from our study show the transgluteal approach had a complication rate of 6% (n=6) at a mean follow-up time of 2.51 years. The most common complication we found was superficial wound infection treated with oral antibiotics (n = 2). We also found only a 1% incidence of periprosthetic fracture (n=1); the literature suggests this could be higher with other approaches, with one study of 3574 patients showing a periprosthetic fracture rate of 9.9% and 8.4% for the posterior and anterior approaches respectively. We report a 1% incidence of DVT (n=1) in our patient cohort with the TGA.

Our study reports a 0% incidence of dislocation and 0% incidence of Trendelenburg gait with the transgluteal approach at short and mid-term follow-up. Traditionally, the posterior approach is thought to have a higher dislocation rate compared to other approaches [23], mitigated with robust soft tissue repair or postoperative hip precautions. A large retrospective study of 13335 patients reported a higher dislocation rate (p=0.026) in the posterior approach (1.1%) versus anterior (0.7%) [24]. Furthermore, two systematic reviews both in excess of 150000 patients support this conclusion [25,26]. A more recent systematic review however found that although the dislocation rate is higher with the posterior approach, this is not statistically significant [27]. Results from our study show a 0% dislocation rate with the transgluteal approach at one year or three years.

Trendelenburg gait can be a result of hip abductor weakness; some studies suggest this is more likely when an approach which disrupts the abductor mechanism has been utilised [28]. A systematic review including 517 patients concluded that the posterior approach confers a risk reduction of Trendelenburg gait postoperatively compared to the lateral approach (OR=0.43, p=0.008) [29].

The transgluteal approach involves disruption of the most distal 1-2 cm of the abductor muscles where the fibres are aligned horizontally. We believe their role is mainly to preserve anterior hip stability rather than to contribute to pelvic stability and the key to preserving abductor function is avoiding disruption to the more superior, oblique fibres which support the pelvis preventing a Trendelenburg gait. The stay stitch used within the cuff may also add to the strength of the abductor repair. We report no incidences of Trendelenburg gait at the one-year follow-up with the TGA.

Cessation of walking aids

Although the use of walking aids may be influenced by pre-existing co-morbidities such as neuropathy or osteoarthritis affecting other joints, it is still incorporated into PROM scoring systems. A search of the literature yielded three studies that reported on time for discontinuing walking aids [30-32]; these three studies showed a statistically significant reduction (p=0.03, p=0.04, p=0.04) in the mean time to the cessation of walking aids in the anterior (33, 23 and 17 days respectively) versus posterior approach (43, 35 and 24 days respectively) [5]. We found cessation of walking aids was 4.86 weeks (34 days) with the transgluteal approach. Further prospective studies comparing cessation of walking aids with a transgluteal approach compared to other approaches would be beneficial.

Radiographic outcomes

Postoperative assessment of radiographic outcomes is important as complications may present asymptomatically [33]. A systematic review and meta-analysis of five studies examined the postoperative implant anteversion and abduction angles of 503 patients for the anterior versus posterior approach [17]. The authors concluded that there was no significant difference (p=0.30) between the two groups with regard to implant anteversion or abduction angles. A similar meta-analysis of 7377 patients found that there was no significant difference (p=0.33) in prosthesis position with either approach [34]. Although radiographic parameters were not formally measured in our study, we report that all patients with the transgluteal approach had satisfactory implant positions at the one-year clinic follow-up, with no wear, loosening, fracture, or prosthetic migration.

Limitations

This study has inherent limitations due to its retrospective design. For example, patient recall of when they ceased to use walking aids may have been affected by recall bias. The OHS results were, however, specific to that point in time and thus not subject to recall bias. The OHS was not performed at specific time points, limiting the ability to assess statistical significance between time periods. The relatively small sample size limits the power of the study. This study also had no parallel control cohort. Finally, the heterogeneity of the operation sub-type (cemented, hybrid, uncemented) may also impact reported outcomes. 

## Conclusions

We report on the results of 100 THAs carried out through a modified transgluteal approach that preserves abductor function and retains the advantage of a low dislocation rate. We report zero cases of dislocation at the one- or three-year mark and no Trendelenburg gait at the one-year follow-up. In hospital LOS, cessation of walking aids and OHS results are comparable to the literature. In our study, 94% of patients had no short to mid-term complications. The mean OHS score was 45.5, indicating satisfactory joint function at a mean time of 972 days post-operatively. Further prospective studies comparing the transgluteal approach to other approaches would be beneficial.
